# The voice of COVID-19: Acoustic correlates of infection in sustained vowelsa)

**DOI:** 10.1121/10.0005194

**Published:** 2021-06-21

**Authors:** Katrin D. Bartl-Pokorny, Florian B. Pokorny, Anton Batliner, Shahin Amiriparian, Anastasia Semertzidou, Florian Eyben, Elena Kramer, Florian Schmidt, Rainer Schönweiler, Markus Wehler, Björn W. Schuller

**Affiliations:** 1 EIHW–Chair of Embedded Intelligence for Health Care and Wellbeing, University of Augsburg, Eichleitnerstrasse 30, 86159 Augsburg, Germany; 2 audEERING GmbH, Friedrichshafener Strasse 1, 82205 Gilching, Germany; 3 Department of Otorhinolaryngology, Phoniatrics and Paediatric Audiology, University Hospital of Schleswig-Holstein, Campus Lübeck, Ratzeburger Allee 160, 23538 Lübeck, Germany; 4 Department of Emergency Medicine and Medicine IV, University Medical Center Augsburg, Stenglinstrasse 2, 86156 Augsburg, Germany

## Abstract

COVID-19 is a global health crisis that has been affecting our daily lives throughout the past year. The symptomatology of COVID-19 is heterogeneous with a severity continuum. Many symptoms are related to pathological changes in the vocal system, leading to the assumption that COVID-19 may also affect voice production. For the first time, the present study investigates voice acoustic correlates of a COVID-19 infection based on a comprehensive acoustic parameter set. We compare 88 acoustic features extracted from recordings of the vowels /i:/, /e:/, /u:/, /o:/, and /a:/ produced by 11 symptomatic COVID-19 positive and 11 COVID-19 negative German-speaking participants. We employ the Mann-Whitney U test and calculate effect sizes to identify features with prominent group differences. The mean voiced segment length and the number of voiced segments per second yield the most important differences across all vowels indicating discontinuities in the pulmonic airstream during phonation in COVID-19 positive participants. Group differences in front vowels are additionally reflected in fundamental frequency variation and the harmonics-to-noise ratio, group differences in back vowels in statistics of the Mel-frequency cepstral coefficients and the spectral slope. Our findings represent an important proof-of-concept contribution for a potential voice-based identification of individuals infected with COVID-19.

## I. INTRODUCTION

In December 2019 and early January 2020, a cluster of pneumonia cases with unknown cause emerged in China's Hubei Province. The pneumonia was found to be caused by a novel coronavirus named *severe acute respiratory syndrome coronavirus 2* (SARS-CoV-2). The disease spread quickly and the first known cases outside of China were identified in mid-January. On 11 February 2020, the World Health Organization (WHO) announced that the disease caused by SARS-CoV-2 would be named COVID-19. A month later, the WHO announced COVID-19 as a pandemic. A year after the emergence of COVID-19, 98 794 942 confirmed cases including 2 124 193 deaths were reported to the WHO (2021).

The severity of COVID-19 is heterogeneous, ranging from asymptomatic infections or mild flu-like symptoms to severe illness and death. Chest CT (Ye *et al.*, 2020), lung ultrasound (Allinovi *et al.*, 2020; Demi, 2020), and post-mortem biopsies (Beigmohammadi *et al.*, 2021; Tian *et al.*, 2020) revealed pathological changes in patients with COVID-19 and suggest that the lung is the organ that is primarily affected by the disease. Common symptoms of COVID-19 include fever, cough, shortness of breath, weakness, muscle pain, loss of taste and/or smell, as well as ear-nose-throat manifestations, sore throat, and headache (Esakandari *et al.*, 2020). Less common ear-nose-throat manifestations of COVID-19 are tonsil enlargement, pharyngeal erythema, nasal congestion, rhinorrhea, and upper respiratory tract infection (El-Anwar *et al.*, 2020). Lechien *et al.* (2020) reported dysphonia for 26.8% of their investigated patients with mild-to-moderate COVID-19 symptoms. The authors further found a greater severity of COVID-19 symptoms in dysphonic patients compared to non-dysphonic patients. In general, a great proportion of the symptoms associated with COVID-19 affect anatomical correlates of speech production. Important components of the vocal system are the lungs and the lower airway producing the airflow, the vocal folds whose vibrations produce the voice sound, and the vocal and nasal tracts modifying the voice source to produce specific phones, cf. Zhang (2016).

Voice changes have been repeatedly reported for a number of diseases related to pathological changes in components of the vocal system. For example, patients with asthma were found to differ from healthy controls in maximum phonation time (MPT), shimmer, harmonics-to-noise ratio (HNR), jitter, fundamental frequency (*f*_o_), frequency of the first vowel formant (*F*_1_), frequency of the second vowel formant (*F*_2_), and frequency of the third vowel formant (*F*_3_) (Balamurali *et al.*, 2020; Dogan *et al.*, 2007; Sonu and Sharma, 2012). Walia and Sharma (2016) found the severity of asthma to be related to jitter [%]. Jitter values derived from recordings of the sustained phonation of the vowel /a:/ were 0.25 for healthy males, 0.41 for males with mild asthma, 0.9 for males with moderate asthma, and 1.83 for males with severe asthma (Walia and Sharma, 2016). Petrović-Lazić *et al.* (2011) reported that jitter, shimmer, *f*_o_ variation, voice turbulence index (VTI), pitch perturbation quotient (PPQ), amplitude perturbation quotient (APQ), and HNR values differed between patients with vocal fold polyps and healthy controls. Type and size of vocal fold polyps were found to have effects on jitter and HNR (Akbari *et al.*, 2018). Male and female patients with unilateral vocal fold paralysis were found to differ from healthy gender-matched controls in jitter, shimmer, HNR, standard deviation of *f*_o,_ and standard deviation of the frequency of *F*_2_ (Jesus *et al.*, 2015). In addition, the males with unilateral vocal fold paralysis differed significantly from the male controls in *F*_1_ and *F*_2_ frequency values as well as in the standard deviation of the frequency of *F*_1_ (Jesus *et al.*, 2015). Segura-Hernández *et al.* (2019) investigated voice characteristics in children with cleft lip and palate before and after speech and language pathology intervention and compared their findings with the voice characteristics of healthy controls. They found that jitter and shimmer were significantly higher in the patients with cleft lip and palate before intervention, whereas the two groups did not differ in these parameters after intervention. In contrast, intervention had no effect on hypernasality, a prominent voice characteristic of patients with cleft lip and palate.

These findings, demonstrating vocal atypicalities in a variety of diseases related to pathological changes in components of the vocal system, lead to the assumption that COVID-19 may be characterised through atypical voice parameters. Characteristic vocal patterns would constitute the starting point for an automatic quick-and-easy-to-apply COVID-19 detection, for example, based on smartphone applications. To date, there is hardly any literature on voice parameters of patients with COVID-19. Recently, Asiaee *et al.* (2020) compared voice samples of a sustained vowel /a:/ produced by Persian speakers with and without COVID-19. They extracted the following eight acoustic parameters: *f*_o_ and its variations (*f*_o_ standard deviation), jitter, shimmer, HNR, difference between the first two harmonic amplitudes (*A*_1_–*A*_2_), MPT, and cepstral peak prominence (CPP). Apart from *f*_o,_ all acoustic parameters were significantly different between the patients with COVID-19 and healthy controls. To the best of our knowledge, voice parameters have not yet been analysed for other vowels and there is no study focusing on the voice of German-speaking patients with COVID-19. The present study aims to provide a deeper insight into voice characteristics of patients with COVID-19 by extracting and comparing a comprehensive set of voice parameters from voice samples of the sustained vowels /i:/, /e:/, /u:/, /o:/, and /a:/ produced by German-speaking symptomatic patients with COVID-19 and healthy controls.

## II. METHODS

For this study, participants were recruited at the University Medical Center Augsburg (six patients with COVID-19, six healthy controls) and via recruitment flyers from the public (five participants with COVID-19, five healthy controls). Taken together, we include a group of 11 adult participants with COVID-19, referred to as +COV, and a group of 11 adult healthy controls, referred to as –COV. The participants of +COV and –COV are gender-matched (4 females and 7 males, respectively) and nearly age-matched (+COV: mean age = 60 years ± 20 years standard deviation; age range = 19–79 years; –COV: mean age = 55 years ± 20 years standard deviation; age range = 24–85 years). All participants of +COV were tested positive for COVID-19 within the last 3 days prior to inclusion into the study; all participants of –COV were tested negative for COVID-19 within the last 3 days prior to inclusion into the study. The participants of –COV had neither symptoms of a cold nor chronic pulmonary or voice diseases. The participants of +COV had mild-to-moderate respiratory symptoms of COVID-19. All participants have German as first language and are residents of Germany or Austria. All participants gave their written informed consent for participation in the study. The study procedures are approved by the study commission of the University Medical Center Augsburg, Germany, as well as by the ethics representative of the University of Augsburg, Germany.

The participants were audio-recorded or recorded themselves while producing the sustained vowels /a:/, /e:/, /i:/, /o:/, /u:/ (order like this), representing phonemes of German standard language. They were instructed to produce each vowel as long as possible and to make a breathing break after each vowel. Recordings were taken in quiet rooms using a smartphone at a distance of approximately 40 cm from the participant's face. In the hospital setting, a Motorola g6 plus smartphone was used. The participants recruited from the public used their own smartphones for the voice recordings.

In a first audio pre-processing step, the recordings are converted into the uniform audio format 16 kHz/16 Bit (single channel) PCM by means of ffmpeg (FFmpeg, 2021). Then, we use audacity (Audacity, 2021) to segment the recordings for all single vowels to be exported as separate audio files for the feature extraction step. The segmented vowels of +COV have a mean duration of 5.9 s ± 3.4 s standard deviation, the segmented vowels of –COV have a mean duration of 12.9 s ± 9.8 s standard deviation.

Acoustic feature extraction is done by means of the open-source toolkit opensmile [audEERING GmbH (2021); Eyben *et al.*, 2013; Eyben *et al.*, 2010] in its current release 3.0.

From each single vowel, we extract the features of the extended Geneva Minimalistic Acoustic Parameter Set (eGeMAPS), representing a compact standard set of 88 acoustic higher-level signal descriptors launched in 2016 by Eyben *et al.* (2016). These higher-level descriptors include statistical functionals, such as arithmetic mean, coefficient of variation, percentiles, etc., computed for the trajectories of a range of acoustic time-, energy-, and/or spectral/cepstral-based low-level descriptors, such as *f*_o,_ Mel-frequency cepstral coefficients (MFCCs), HNR, jitter, or shimmer. While being a comparably small set among the available opensmile standard sets, the features of the eGeMAPS were carefully selected by a consortium of engineers, linguists, phoneticians, and clinicians based on their theoretical and practical value for computational voice analysis tasks including clinical applications (Eyben *et al.*, 2016).

We apply the Mann-Whitney U test (group- and vowel-wise feature values are not normally distributed) to analyse the distributions of the extracted acoustic features for differences between +COV and –COV. On the one hand, this is done separately for each vowel. On the other hand, we analyse the combination of the front vowels /i:/ and /e:/, of the back vowels /u:/ and /o:/, as well as of all vowels. To identify the most important acoustic features in either constellation to distinguish between +COV and –COV, we finally rank the acoustic features according to the effect size *r* as being the absolute value of the correlation coefficient [*z*-value divided by the square root of the number of samples (Rosenthal, 1994)]. Top features are defined to have an effect size *r *> * *0.3. As null-hypothesis testing with *p*-values as decisive criterion has been repeatedly criticised [see the statement by the American Statistical Association (Wasserstein and Lazar, 2016)], we here report two-sided *p*-values together with a Bonferroni-corrected significance level as additional descriptive measures and do not employ them for accepting or rejecting a null-hypothesis.

## III. RESULTS

**FIG. 1.**
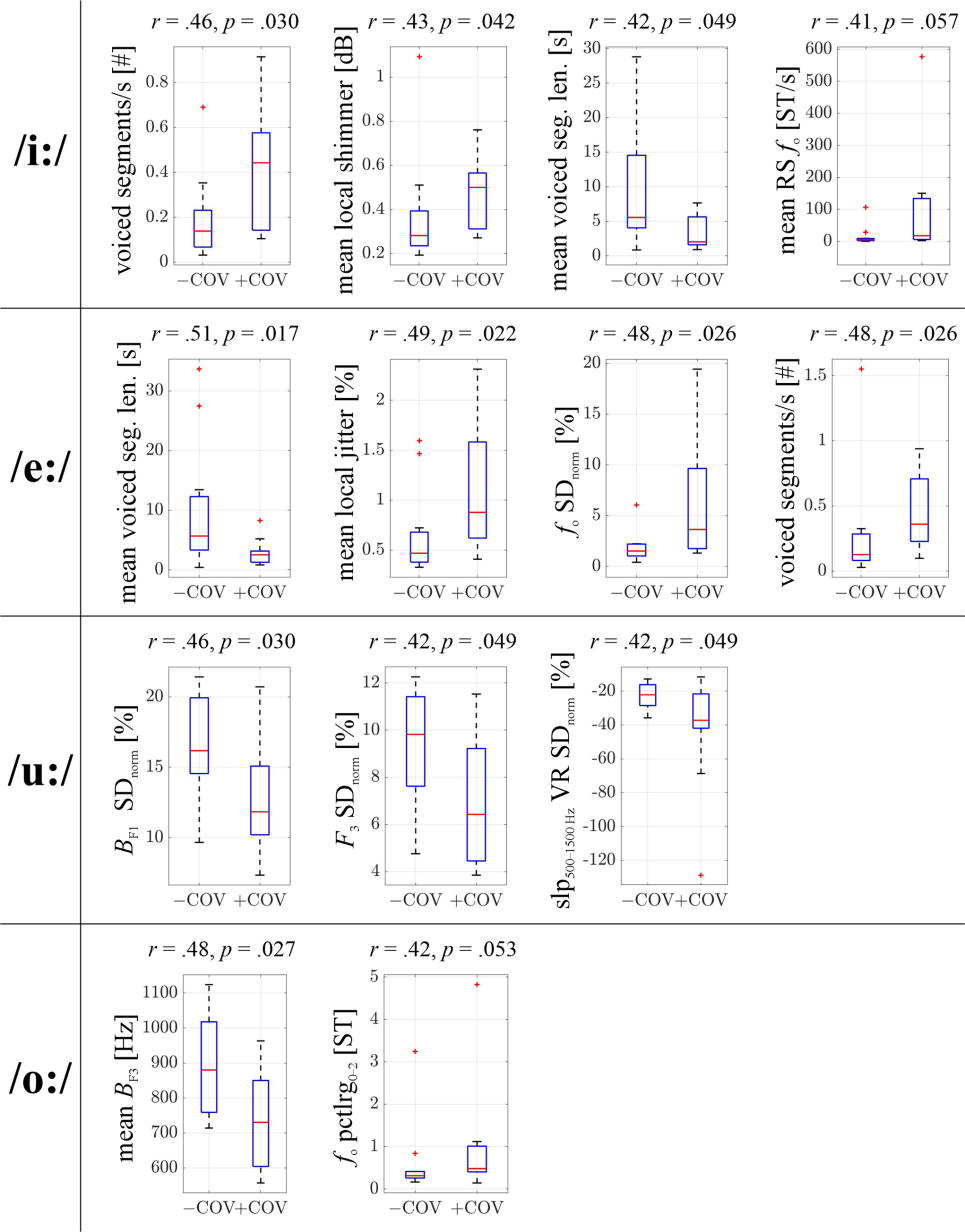
(Color online) Vowel-wise acoustic feature comparisons between COVID-19 negative (–COV) and COVID-19 positive (+COV) participants in form of boxplots for features with a differentiation effect *r *> 0.4 ordered from left to right according to a decreasing *r*, respectively. The effect size *r* as well as the *p*-value of the Mann-Whitney U difference test are given above each boxplot. *r* is rounded to two decimal places. *p* is rounded to three decimal places. Level of significance after Bonferroni correction: 7 × 10^−4^. Outliers (marked with red plus symbols) are defined as values that are more than 1.5 times the interquartile range away from the bottom or top of the respective box. # = number of, *B_F_*_1,3_ = bandwidth of first and third vowel formant, *f*_o_ = fundamental frequency, *F*_3_ = frequency of third vowel formant, len. = length, pctlrg = percentile range, RS = rising slope, seg. = segment, ST = semitone from 27.5 Hz, SD_norm_ = standard deviation normalised by the arithmetic mean (coefficient of variation), slp = slope, VR = voiced regions.

We display the respective top acoustic features, i.e., features differing between +COV and –COV with an effect size *r *>* *0.3, for the single-vowel scenarios as well as for the vowel combination scenarios in Tables I and II. Altogether, 72 top features are identified across the 8 scenarios. Based on a significance level of 0.05, the Bonferroni-corrected significance level for 72 repeated measurements is 7 × 10^−4^. Additionally, we present boxplots for all features with an effect size *r *>* *0.4 in the single-vowel examinations in Fig. 1. The mean voiced segment length as well as the number of voiced segments per second represent the features that differ most between +COV and –COV in terms of effect size when combining recordings of all vowels, i.e., the test scenario with the largest sample size. The corresponding *p*-values—sample-size dependent—are below the Bonferroni-corrected significance level. Further features with prominent group differences across vowels and vowel constellations are bandwidth statistics of the third vowel formant and local shimmer. Additionally, group differences in (a) front vowels are reflected in *f*_o_-related statistics and the coefficient of variation of the HNR, and in (b) back vowels in statistics related to the first two MFCCs and in the coefficient of variation of the spectral slope 500–1500 Hz in voiced regions.

**TABLE I.** Vowel-wise acoustic features with a differentiation effect *r *> 0.3 between COVID-19 negative and COVID-19 positive participants, ranked according to the effect size *r*. *r* is rounded to two decimal places. *p*-values of the underlying Mann-Whitney U tests rounded to three decimal places are given as well. Level of significance after Bonferroni correction: 7 × 10^−4^. *A*_1_ = relative amplitude of first harmonic, *A*_F3_ = amplitude of third vowel formant, *B_F_*_1,2,3_ = bandwidth of first, second, and third vowel formant, *f*_o_ = fundamental frequency, *F*_3_ = frequency of third vowel formant, HNR = harmonics-to-noise ratio, MFCC_1,2,4_ = first, second, and fourth Mel-frequency cepstral coefficient, pctl = percentile, pctlrg = percentile range, SD_norm_ = standard deviation normalised by the arithmetic mean (coefficient of variation), VR = voiced regions.

Vowel	Rank	Feature	*r*	*p*
/i:/	1	voiced segments per second	0.46	0.030
	2	mean local shimmer	0.43	0.042
	3	mean voiced segment length	0.42	0.049
	4	mean rising slope *f*_o_	0.41	0.057
	5	rising slope *f*_o_ SD	0.39	0.066
	6	harmonic difference *A*_1_–*A*_F3_ SD_norm_	0.38	0.076
	7	MFCC_2_ VR SD_norm_	0.36	0.088
	8	HNR SD_norm_	0.35	0.101
	9	*B*_F3_ SD_norm_	0.34	0.115
	10	voiced segment length SD	0.33	0.118
	11	*B*_F2_ SD_norm_	0.32	0.131
	12	MFCC_1_ VR SD_norm_	0.31	0.149
/e:/	1	mean voiced segment length	0.51	0.017
	2	mean local jitter	0.49	0.022
	3	*f*_o_ SD_norm_	0.48	0.026
	4	voiced segments per second	0.48	0.026
	5	HNR SD_norm_	0.39	0.066
	6	*B*_F3_ SD_norm_	0.38	0.076
	7	MFCC_1_ VR SD_norm_	0.34	0.115
/u:/	1	*B*_F1_ SD_norm_	0.46	0.030
	2	*F*_3_ SD_norm_	0.42	0.049
	3	slope_500–1500Hz_ VR SD_norm_	0.42	0.049
	4	mean MFCC_1_	0.39	0.066
	5	MFCC_4_ SD_norm_	0.39	0.066
	6	MFCC_1_ VR SD_norm_	0.39	0.066
	7	mean MFCC_1_ VR	0.38	0.076
	8	loudness pctl_20_	0.36	0.088
	9	mean *B*_F3_	0.34	0.115
	10	slope_0–500Hz_ VR SD_norm_	0.34	0.115
/o:/	1	mean *B*_F3_	0.48	0.027
	2	*f*_o_ pctlrg_0–2_	0.42	0.053
	3	slope_500–1500Hz_ VR SD_norm_	0.39	0.073
	4	mean MFCC_1_ VR	0.39	0.073
	5	mean local shimmer	0.35	0.113
	6	*F*_3_ SD_norm_	0.35	0.113
	7	falling slope loudness SD	0.33	0.130
	8	mean MFCC_1_	0.33	0.130
	9	rising slope loudness SD	0.32	0.149
	10	mean MFCC_2_ VR	0.32	0.149
/a:/	1	mean voiced segment length	0.39	0.066
	2	mean loudness	0.38	0.076
	3	mean MFCC_2_ VR	0.38	0.076
	4	loudness pctl_50_	0.35	0.101
	5	spectral flux SD_norm_	0.35	0.101
	6	mean MFCC_2_	0.34	0.115
	7	mean Hammarberg index VR	0.32	0.131
	8	loudness pctl_80_	0.31	0.149
	9	slope_500–1500Hz_ VR SD_norm_	0.31	0.149
	10	loudness peaks per second	0.31	0.149
	11	voiced segments per second	0.31	0.149

**TABLE II.** Acoustic features with a differentiation effect *r *>* * 0.3 between COVID-19 negative and COVID-19 positive participants, ranked according to the effect size *r* for the combination of the front vowels /i:/ and /e:/, the back vowels /u:/ and /o:/, and all vowels. *r* is rounded to two decimal places. *p*-values of the underlying Mann-Whitney U tests rounded to three decimal places are given as well. Level of significance after Bonferroni correction: 7 × 10^−4^. *A*_1_ = relative amplitude of first harmonic, *A*_F3_ = amplitude of third vowel formant, *B_F_*_1,2,3_ = bandwidth of first, second, and third vowel formant, *f*_o_ = fundamental frequency, *F*_3_ = frequency of third vowel formant, HNR = harmonics-to-noise ratio, MFCC_1,2_ = first and second Mel-frequency cepstral coefficient, SD_norm_ = standard deviation normalised by the arithmetic mean (coefficient of variation), VR = voiced regions.

Vowel	Rank	Feature	*r*	*p*
/i:/ ∪/e:/	1	voiced segments per second	0.48	0.002
	2	mean voiced segment length	0.47	0.002
	3	HNR SD_norm_	0.37	0.014
	4	*B*_F3_ SD_norm_	0.37	0.014
	5	mean local shimmer	0.35	0.022
	6	*f*_o_ SD_norm_	0.32	0.036
	7	harmonic difference *A*_1_–*A*_F3_ SD_norm_	0.31	0.038
	8	MFCC_1_ VR SD_norm_	0.31	0.038
	9	voiced segment length SD	0.31	0.039
/u:/ ∪/o:/	1	mean *B*_F3_	0.42	0.006
	2	mean MFCC_1_ VR	0.49	0.009
	3	*B*_F1_ SD_norm_	0.38	0.012
	4	*F*_3_ SD_norm_	0.38	0.014
	5	mean MFCC_1_	0.36	0.018
	6	MFCC_1_ VR SD_norm_	0.34	0.026
	7	mean local shimmer	0.33	0.032
	8	*B*_F2_ SD_norm_	0.32	0.036
	9	slope_500–1500Hz_ VR SD_norm_	0.31	0.040
	10	mean voiced segment length	0.31	0.041
	11	mean MFCC_2_ VR	0.30	0.048
/i:/ ∪/e:/ ∪/u:/ ∪/o:/ ∪/a:/	12	mean voiced segment lengthvoiced segments per second	0.390.38	4 × 10^−5^8 × 10^−5^

## IV. CONCLUSIONS

**FIG. 2.**
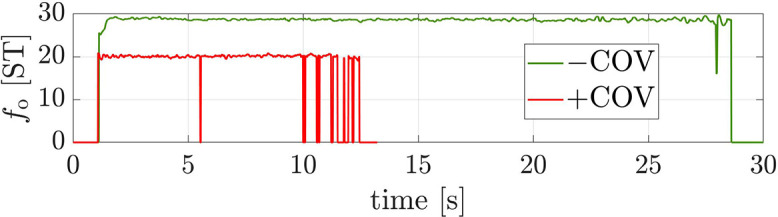
(Color online) Fundamental frequency (*f*_o_) trajectories for the production of the sustained vowel /e:/ of a 68-year-old male COVID-19 negative participant (–COV, green line) and a 54-year-old male COVID-19 positive participants (+COV, red line). ST = semitone from 27.5 Hz.

In this study, we acoustically analysed sustained vowels produced by participants with a COVID-19 infection and a group of healthy controls. We identified a number of acoustic features to moderately differ between the two groups. We found that +COV produced a higher number of voiced segments per second at a shorter mean voiced segment length as compared to –COV. As participants were instructed to produce sustained vowels, i.e., with a continuous phonation over a certain time, this finding may indicate discontinuities in the pulmonic airstream in COVID-19 infected participants leading to sporadic, unintended interruptions of phonation. The prototypical *f*_o_ contours for +COV vs –COV in Fig. 2 demonstrate that +COV tends much more towards irregular phonation than –COV. Irregular phonation is shown by the frequent “breakdowns” of the *f*_o_ curve, especially towards the end of phonation: Here, the opensmile extraction algorithm cannot find a (meaningful) *f*_o_ value and outputs zero. In consequence, there are more voiced segments per second for +COV and, concomitantly, voiced segments of +COV are shorter. This can be conceived as a sort of epiphenomenon of irregular phonation, indicating weakened tension of the vocal folds and less control of phonation. The shorter mean voiced segment length is further related to the shorter overall vocalisation duration of +COV (see Sec. II). Voiced segments per second and mean voiced segment length as sort of (overall) duration measures are important in front vowels—separately and taken together, not amongst the most important in back vowels, and again amongst the important ones in /a:/. They turn out to be most important across all vowels. With a caveat, this might be due to the overall effort that is lower for back vowels whose tongue position is closer to the [ə] (schwa). For /a:/, both duration and loudness features are relevant. This vowel has the largest degree of opening, the largest airflow, the greatest intensity, and the greatest extent of articulatory movements; it is notoriously prone to irregular phonation (laryngealisation) (Batliner *et al.*, 2007). Thus, for COVID-19 positive patients, sensory control over articulation might be more limited in the production of /a:/.

Asiaee *et al.* (2020) partly analysed the same acoustic features as used in our study. Among these, the *f*_o_ standard deviation, jitter, shimmer, and the HNR were found to be different between COVID-19 positive and COVID-19 negative participants when comparing voice samples of a sustained vowel /a:/. In our study, these features are not among the most important ones to differentiate the groups in the sustained vowel /a:/. However, the normalised *f*_o_ standard deviation and local jitter turned out to be relevant for group differentiation in the front vowel /e:/, the normalised HNR standard deviation in the front vowels /i:/ and /e:/, and local shimmer in the vowels /i:/ and /o:/. Divergent findings between our study and the study by Asiaee *et al.* (2020) may result from the fact that the participants of the latter were Persian speakers, whereas participants in our study are German speakers. All things considered, findings by Asiaee *et al.* (2020) as well as our findings pointing to voice acoustic correlates of a COVID-19 infection across the frequency (e.g., *f*_o,_ formant frequencies, jitter), energy (e.g., shimmer, HNR), and in our study also spectral/cepstral (e.g., MFCCs, slope, harmonic difference) domains suggest that a COVID-19 infection may not be characterisable by a single feature, but by a combination of selected candidate features tied to specific phonation tasks.

Acoustic analysis in this work is based on a compact standard feature set designed for a variety of computational voice analysis tasks also including tasks in clinical context. Starting from the gained knowledge about each feature's relevance for reflecting vocal differences between COVID-19 positive and COVID-19 negative speakers, future work should additionally focus on specific clinical speech parameters that allow for interpretations from a voice-physiological point of view, such as the glottal-to-noise excitation (GNE) ratio (Michaelis *et al.*, 1998; Michaelis *et al.*, 1997).

The present study can be regarded as an initial proof-of-concept investigation for a voice-based differentiation between individuals with and without a COVID-19 infection in a real-world context. Thus, it motivates the implementation of automatic voice-based COVID-19 detection applications, which will likely be developed for smartphones. This is why we accept limitations related to data acquisition “in the wild” using smartphones as compared to data acquisition in a standardised laboratory setting. In order to maximise data quality and minimise bias, we instructed all participants to take the recordings in a quiet room at a predefined distance from the face. Moreover, variation with respect to the used smartphone (microphone sensitivity, frequency response, etc.), used smartphone recording app (automatically set filters, audio codec/compression, etc.), exact microphone-mouth distance and angle, room acoustics, etc., is present in both +COV and –COV. A bias towards one of the investigated groups is thus unlikely. Another data acquisition-related limitation of our study is that some participants obviously did not follow the instruction to produce each vowel as long as possible. This applies both to participants from +COV and –COV. Although differences in the produced vowel duration have to be interpreted with caution, it is interesting that participants of –COV on average vocalised more than twice as long as participants of +COV. This might indicate that producing sustained vowels is more uncomfortable for participants with a COVID-19 infection compared to healthy controls. Due to the inhomogeneous realisation of the sustained vowel task with regard to phonation duration among the participants of this study, we here investigated acoustic features extracted from the entire vowels. However, we suggest an additional analysis of specific vowel parts, such as the first third including the vowel onset, the middle third, and the last third including the vowel offset, for future work. A further limitation of our study is the relatively small sample size. Moreover, –COV consists of healthy speakers only, i.e., speakers without any symptoms of a cold, whereas +COV only includes patients with mild-to-moderate flu-like symptoms. To evaluate whether there are voice parameters specific for a COVID-19 infection, future studies need to include a considerable amount of patients with COVID-19 who do not show respiratory or ear-nose-throat symptoms and COVID-19 negative participants with cold-like symptoms. Moreover, as some of the acoustic features we show to be important for a differentiation between COVID-19 positive and COVID-19 negative participants were also reported to be relevant for differentiating between patients with asthma and healthy controls (Dogan *et al.*, 2007; Sonu and Sharma, 2012; Walia and Sharma, 2016), it is highly important for future studies to include patients with asthma and other chronically ill patients in a control group.

Despite the limitations, our study can be regarded as a first step towards unravelling the complex acoustic fingerprint of COVID-19 and as an important proof-of-concept achievement for future voice-based viral infection identification applications. A re-validation of our findings based on a larger and more heterogeneous sample is warranted.
